# Design of Antioxidant Nanoparticle, which Selectively Locates and Scavenges Reactive Oxygen Species in the Gastrointestinal Tract, Increasing The Running Time of Mice

**DOI:** 10.1002/advs.202301159

**Published:** 2023-08-01

**Authors:** Takuto Toriumi, Hajime Ohmori, Yukio Nagasaki

**Affiliations:** ^1^ Department of Materials Science Faculty of Pure and Applied Sciences University of Tsukuba 1‐1‐1 Tennoudai Tsukuba Ibaraki 305‐8573 Japan; ^2^ University of Tsukuba 1‐1‐1 Tennoudai Tsukuba Ibaraki 305‐8573 Japan; ^3^ Faculty of Business Information Sciences Jobu University Toyazukamachi 634‐1 Isesaki Gunma 372‐8588 Japan; ^4^ Master's School of Medical Sciences Graduate School of Comprehensive Human Sciences University of Tsukuba Tennoudai 1‐1‐1 Tsukuba Ibaraki 305‐8573 Japan; ^5^ Center for Research in Radiation, Isotope and Earth System Sciences (CRiES) University of Tsukuba Tennoudai 1‐1‐1 Tsukuba Ibaraki 305‐8573 Japan; ^6^ Department of Chemistry Graduate School of Science The University of Tokyo Hongo 7‐3‐1 Bunkyo‐ku Tokyo 113‐8654 Japan

**Keywords:** exercise‐induced gastrointestinal syndrome, high‐intensity running, leaky gut, polymeric nanoparticle antioxidants, reactive oxygen species

## Abstract

Excess reactive oxygen species (ROS) produced during strong or unfamiliar exercise cause exercise‐induced gastrointestinal syndrome (EIGS), leading to poor health and decreased exercise performance. The application of conventional antioxidants can neither ameliorate EIGS nor improve exercise performance because of their rapid elimination and severe side effects on the mitochondria. Hence, a self‐assembling nanoparticle‐type antioxidant (RNP^O^) that is selectively located in the gastrointestinal (GI) tract for an extended time after oral administration is developed. Interestingly, orally administered RNP^O^ significantly enhances the running time until exhaustion in mice with increasing dosage, whereas conventional antioxidants (TEMPOL) tends to reduce the running time with increasing dosage. The running (control) and TEMPOL groups show severe damage in the GI tract and increased plasma lipopolysaccharide (LPS) levels after 80 min of running, resulting in fewer red blood cells (RBCs) and severe damage to the skeletal muscles and liver. However, the RNP^O^ group is protected against GI tract damage and elevation of plasma LPS levels, similar to the nonrunning (sedentary) group, which prevents damage to the whole body, unlike in the control and TEMPOL groups. Based on these results, it is concluded that continuous scavenging of excessive intestinal ROS protects against gut damage and further improves exercise performance.

## Graphical abstract

Antioxidant nanoparticle (RNP^O^), which stays specifically in the gastrointestinal tract without disturbance of mitochondrial dysfunction, is developed. RNP^O^ improves exercise performance in a dose‐dependent manner. Because RNP^O^ scavenges reactive oxygen species (ROS) in the gastrointestinal tract and inhibits leaky gut‐induced decrease in red blood cells and damage in whole body.

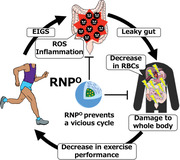

## 1 Introduction

Exercise is good for health, and many people routinely perform exercise.^[^ ^1^ ^]^ Aerobic exercise can be expected to improve the function of the respiratory and cardiovascular systems in addition to burning body fat. Therefore, exercise can be anticipated to improve body condition. However, increasing reports indicate that excessive or unfamiliar exercise is detrimental to health and decreases exercise performance.^[^ ^2^, ^3^ ^]^ During strong and/or long‐term exercise, a large amount of oxygen is required, and a considerable number of reactive oxygen species (ROS), such as superoxide, hydrogen peroxide, and hydroxy radicals, are produced simultaneously.^[^ ^4^ ^]^ Although ROS and antioxidant capacity are moderately balanced under normal conditions in the body, excessive ROS production during aerobic exercise cannot be controlled by the endogenous antioxidant system.^[^ ^5^, ^6^ ^]^ Therefore, overproduced ROS react with body components, such as lipids, proteins, and DNA, causing tissue degeneration and damage (so‐called oxidative stress), leading to poor health and reduced exercise performance.^[^ ^6^, ^7^ ^]^

Intake of conventional antioxidants is anticipated to counteract the harmful effects of ROS overproduction to improve health and exercise performance.^[^ ^8^ ^]^ However, conventional antioxidants are reported to be ineffective at improving health and exercise performance and may cause damage to the body.^[^ ^9^, ^10^ ^]^ For example, Buchman et al. reported that premarathon running vitamin E supplementation did not affect performance, intestinal injury, or severity of postmarathon running gastrointestinal complaints.^[^ ^11^ ^]^ Why does the elimination of ROS by traditional antioxidants cause adverse effects? Most traditional antioxidants are low molecular weight (LMW) antioxidants, which diffuse nonspecifically and are eliminated rapidly after administration.^[^ ^12^ ^]^ Therefore, LMW antioxidants cannot effectively remove the ROS generated during long‐term exercise. Furthermore, because LMW antioxidants are readily taken up by normal cells, they also remove intracellular ROS, which are required for energy production, resulting in imbalanced cellular homeostasis, including mitochondrial dysfunction.^[^ ^13^, ^14^ ^]^

To solve these problems of LMW antioxidants, it is necessary to improve their bioavailability and reduce their adverse effects, especially dysfunction in normal cells. Recently, we have been developing self‐assembling nanoparticle‐type antioxidants (RNPs) to address the above‐mentioned requirements.^[^ ^15^, ^16^, ^17^, ^18^, ^19^, ^20^, ^21^, ^22^, ^23^, ^24^, ^25^, ^26^, ^27^ ^]^ 2,2,6,6‐Tetramethylpiperidine‐1‐oxyl free radical (TEMPO), one of the strongest antioxidants, was covalently conjugated as a side chain of the repeating units of the hydrophobic segments in amphiphilic block copolymers, which spontaneously self‐assemble into core‐shell type polymer micelles in aqueous media.^[^ ^15^, ^16^, ^28^ ^]^ Poly(ethylene glycol) (PEG) was employed as the hydrophilic segment of the block copolymer, which formed the micelle shell. The PEG shell provides biocompatibility and dispersion stability to nanoparticles under biological conditions, prolonging the bioavailability of RNPs compared with that of LMW antioxidants.^[^ ^17^, ^18^ ^]^ RNPs with a size of several tens of nanometers show reduced internalization into normal cells, avoiding the disruption of intracellular redox homeostasis, which is inevitable with LMW antioxidants.^[^ ^19^, ^20^ ^]^ As RNPs significantly suppressed their unwanted adverse effects, they showed remarkable therapeutic efficiency against several oxidative‐related diseases such as ulcerative colitis, cancer, renal, cerebral, and cardiac ischemia‐reperfusion injuries, Alzheimer's disease, and so on.^[^ ^21^, ^22^, ^23^, ^24^, ^25^, ^26^ ^]^

As stated in the first paragraph of the Introduction, ROS overproduction during exercise is a crucial target for controlling performance and body damage. Therefore, we applied our nanoparticle‐type antioxidant (RNP^N^, a pH‐sensitive RNP, wherein the nanoparticle disintegrates under acidic conditions to expose the antioxidant TEMPO outside) to high‐intensity exercise performance in rats.^[^ ^27^ ^]^ Rats in the control group ran for an average of 60 min on a treadmill at 40 m/min, whereas rats ran for an average of 90 min under the same conditions after the subcutaneous administration of RNP^N^. RNP^N^‐untreated rats showed a significant decrease in red blood cells (RBC) after running, whereas RNP^N^‐treated rats did not, indicating that RNP^N^ effectively showed antioxidant effects in the bloodstream and protected against oxidative damage to RBCs during running. To the best of our knowledge, this study was the first to report that nanoparticle‐type antioxidants reduce exercise‐oriented damage caused by ROS and significantly improve exercise performance by reducing side effects and prolonging bioavailability.

Recently, we investigated the effect of orally administered RNP^N^ on exercise performance. After RNP^N^ was administered orally to rats, a similar tendency as that with subcutaneous administration was observed, viz., the average running time extended by 50% at a dose of ≈2.5 mmol kg^−1^ weight at 40 m min^−1^. As RNP^N^ disintegrated in the acidic stomach, followed by internalization into blood circulation from the intestine,^[^ ^17^ ^]^ we interpreted that the orally administered RNP^N^ worked similarly to its subcutaneous injection, and protected RBCs against ROS attack in the bloodstream. We already confirmed that RNP^N^ uptake into the bloodstream was only a small percentage of the orally administered dose^[^ ^26^ ^]^; however, we could not understand why a similar effect was observed on running performance (See Figure S3, Supporting Information). As we have not answered this question yet, we have not published these data. Notably, more than 90% of RNP^N^ remains in the gastrointestinal (GI) tract after its oral administration.^[^ ^26^ ^]^

In recent years, exercise‐induced gastrointestinal syndrome (EIGS) has become a severe problem during high‐intensity exercise.^[^ ^29^, ^30^ ^]^ Various causes have been reported for this syndrome. For example, Costa et al. reported that heat stress and blood flow redistribution (i.e., changes in distribution from organs to peripheral tissues) caused by exercise results in strong inflammation in the GI tract, which induces EIGS.^[^ ^31^, ^32^ ^]^

**Scheme 1:**
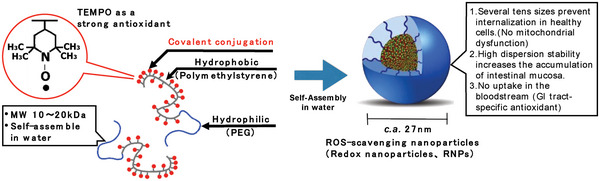
Schematic illustration of RNP^O^.

We hypothesized here that more than 90% of RNP^N^ retained in the GI tract after oral administration might help prevent EIGS and improve exercise performance. In this study, we prepared and evaluated a different type of antioxidant nanoparticle, RNP^O^, which has a non‐pH‐triggered disintegration characteristic that stays specifically in the GI tract after oral administration (no internalization in the bloodstream) to investigate the impact of antioxidant capacity in the GI tract on exercise performance (**Scheme** **1**). RNP^O^ has been confirmed to stay in the GI tract specifically for more than 24 h without leaking into the blood.^[^ ^18^, ^21^ ^]^ Interestingly, orally administered RNP^O^ improved exercise performance in a dose‐dependent manner. In addition, RNP^O^ inhibited the dysfunction of intestinal tissues, RBCs, skeletal muscle, liver, and kidneys, even though RNP^O^ was only retained in the GI tract. Therefore, we suggest a mechanism by which effective removal of ROS in the GI tract by RNP^O^ ameliorates EIGS, suppresses the induction of systemic dysfunction, and improves exercise performance.

## 2 Results

### 2.1 Preparation and Characterization of RNP^O^

Non‐pH‐sensitive nitroxide radical‐containing nanoparticles (RNP^O^) were prepared by dissolving methoxy‐poly(ethylene glycol)‐*b*‐poly[4‐(2,2,6,6‐tetramethylpiperidine‐1‐oxyl)oxymethylstyrene] (MeO‐PEG‐*b*‐PMOT) in *N,N*‐dimethylformamide (DMF), followed by dialysis against distilled and deionized (dd) water through a semi‐permeable membrane. The ^1^H‐NMR spectrum, gel permeation chromatography (GPC) curve, and electron spin resonance (ESR) spectrum of MeO‐PEG‐*b*‐PMOT are shown in Figure S1A–D, Supporting Information. MeO‐PEG‐*b*‐PMOT was confirmed to contain 18 TEMPO units in its PMOT segment. Compared to the triplet signal of the 4‐hydroxy‐2,2,6,6‐tetramethylpiperidine‐1‐oxyl free radical (TEMPOL) in ESR spectrum, MeO‐PEG‐*b*‐PMOT in dimethyl sulfoxide (DMSO) showed a broader singlet signal, indicating the neighboring effect of distance between nitroxide radicals introduced into the side chain of the repeating units in the polymer (Figure S1D, Supporting Information). The RNP^O^ self‐assembled into a core‐shell type nanoparticle structure by the hydrophobic‐hydrophilic balance of the amphiphilic polymer MeO‐PEG‐*b*‐PMOT. The ESR spectrum and the dynamic light scattering (DLS) measurements of RNP^O^ are shown in Figure S2A,B, Supporting Information. The results confirmed that the average hydrodynamic diameter of RNP^O^ in dd water was ≈27 nm. The ESR spectrum of RNP^O^ showed a much broader singlet signal (Figure S2C, Supporting Information), suggesting the confinement of TEMPO molecules in the core of the nanoparticle, in addition to the neighboring effect of the adjacent nitroxide radicals on the repeating units in the polymer.

### 2.2 RNP^N^ Enhances Running Time Until Exhaustion

As described in the introduction, orally administered RNP^N^ greatly enhanced exercise performance, in contrast to that with LMW antioxidants (Figure S3, Supporting Information). For example, the all‐out time of the RNP^N^‐administered group increased by almost 50% compared with that of the control group, whereas the TEMPOL‐administered group did not increase its time at all. This suggests that RNP^N^, which was taken up into the bloodstream at a small percentage upon oral administration, protected RBCs from oxidative damage, similar to that with subcutaneous injection, as shown in our previous paper.^[^ ^17^, ^26^, ^27^ ^]^ It is somewhat curious that only a few % of the administered RNP^N^ in the bloodstream worked similarly to the subcutaneously administered RNP^N^ shown in the previous paper.^[^ ^26^, ^27^ ^]^ Therefore, we hypothesized that more than 90% of RNP^N^, which is retained in the GI tract after oral administration, might affect exercise performance.

### 2.3 RNP^O^ Enhances the Running Time Until Exhaustion in a Dose‐Dependent Manner

As stated above, we hypothesized that ≈90% of RNP^N^ retained in the GI tract might contribute to improving exercise performance. We have already shown that non‐pH‐sensitive nitroxide radical‐containing nanoparticles (RNP^O^) are not internalized in the bloodstream and are retained in the GI tract after oral administration.^[^ ^18^, ^21^ ^]^ Therefore, we investigated the effects of ROS scavenging in the GI tract on exercise performance. Mice were subjected to high‐intensity running for investigating the effect of RNP^O^ on exercise performance. Antioxidants were administered orally 30 min before the test. The mice then ran exhaustively on a treadmill (all‐out running experiment at 28 m min^−1^).

**Figure 1:**
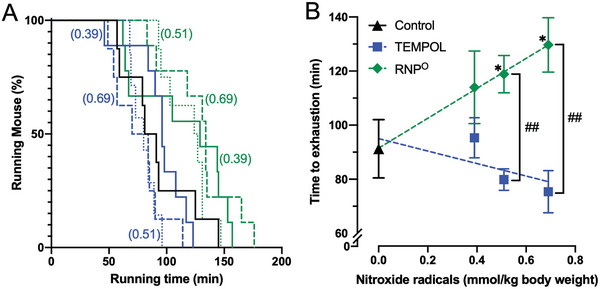
Results of the all‐out running test. A) All‐out time for each mouse. The number in parentheses denotes the dose of TEMPO in 10^−2^ mmol kg^−1^ body weight (Black: Control, 0 mmol nitroxide radicals kg^−1^ body weight, Blue: TEMPOL, Green: RNP^O^). B) Effect of dose dependency on the average all‐out time for the obtained data in Figure 1A. Mice were forced to run until they reach their limit and were no longer able to move forward. TEMPOL was administered at 0.39–0.69 mmol kg^−1^. RNP^O^ was administered at the same dose as the TEMPOL group (0.39–0.69 mmol‐TEMPO kg^−1^, 200–400 mg‐RNP^O^ kg^−1^). Data are expressed as the mean ± standard error of the mean (SEM) (*n* = 7–9). *p < 0.05 versus Control (0 mmol nitroxide radicals kg^−1^ body weight in the graph), ##*p* < 0.01 versus within same concentration between the TEMPOL and RNP^O^ groups.

As shown in **Figure** **1**A, the control group (running after water administration) ran for ≈55–145 min, with an average of 91 min. In the TEMPOL group, the average all‐out running time tended to decrease as the dose increased. In contrast to the TEMPOL group, the RNP^O^ group showed a dose‐dependent increase in the all‐out running time (Figure 1B). Pearson's correlation coefficient confirmed the dose‐dependency (RNP^O^: *r* = 0.4246, *p* < 0.05; TEMPOL: *r* = −0.2548, not significant). The all‐out running time was prolonged with a 40% statistical significance over the average running time at the highest dose (69 × 10^−2^ mmol‐TEMPO/kg‐body weight) compared to that in the control group.

### 2.4 RNP^O^ Inhibits the Intestinal Damage, Inflammation, and Oxidative Stress Caused by High‐Intensity Running

**Figure 2:**
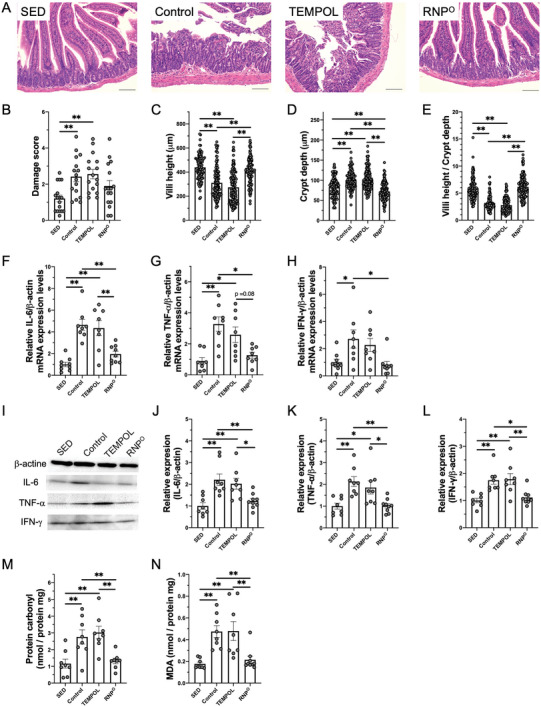
Suppression of high‐intensity running‐induced GI tract damage by RNP^O^. A) Representative histopathological sections of duodenum tissues stained with H&E, scale bar = 100 µm. B) Damage score in the duodenum after rest or high‐intensity running on 80 min (*n* = 16–17). C) Villi height (*n* = 103–131), D) Crypt depth (*n* = 103–131), E) The villus height/crypt depth ratio after rest or high‐intensity running for 80 min. F‐H) Gene expression of inflammatory cytokines in the small intestine after rest or high‐intensity running for 80 min, F) Interleukin‐6 (IL‐6) (*n* = 8–9), G) Tumor necrosis factor alpha (TNF‐α (*n* = 7–8), H) Interferon‐gamma (IFN‐γ (*n* = 8–9). I‐L) Protein expression of inflammatory cytokines in the small intestine after rest or high‐intensity running for 80 min, I) Immunoblots of β‐actin, IL‐6, TNF‐α, and IFN‐γ, J) IL‐6 (*n* = 8–9), K) TNF‐α (*n* = 8–9), L) IFN‐γ (*n* = 8–9). M, N) Oxidative stress levels in the small intestine after rest or high‐intensity running for 80 min, M) Protein carbonyl (*n* = 8), N) Malondialdehyde (MDA) (*n* = 8–9). TEMPOL was administered at 0.69 mmol kg^−1^. RNP^O^ was administered at the same dose as the TEMPOL group (0.69 mmol‐TEMPO kg^−1^, 400 mg‐RNP^O^ kg^−1^). Data are expressed as the mean ± SEM (*p < 0.05, **p < 0.01).

As stated above, orally administered RNP^O^ was selectively retained in the GI tract and clearly improved the running performance. We evaluated small intestinal conditions to investigate the effect of RNP^O^ on intestinal damage by high‐intensity running. **Figure** **2**A–E shows the histological analysis of the duodenum after 80 min of running at 28 m min^−1^ on the treadmill. From the histological analysis of the duodenum stained with hematoxylin and eosin (H&E) shown in Figure 2A, both the running group (control) and the TEMPOL‐administered group showed collapse of intestinal structure as well as a significant decrease in villus length, indicating severe damage to the small intestine. The intestinal damage score increased in both groups (Figure 2B). These phenomena were in sharp contrast to those in the sedentary (nonrunning, SED) group. Compared to the TEMPOL group, the RNP^O^ group did not show significant damage in the histological assessment, which did not increase the intestinal damage score. The intestinal damage score was originally employed in the heat stroke model; therefore, assessing the intestinal damage from exercise, which is less intense than heat stroke, might be difficult. Thus, we measured villus length, crypt depth, and the ratio of these measurements to assess damage to the small intestine. As intestinal damage results in shorter villi and deeper crypts, these lengths and ratios indicate damage to the GI tract. As shown in Figure 2C–E, villus lengths decreased and crypt depths increased in both the control and TEMPOL groups compared to those in the SED group. However, the RNP^O^ group did not show any change in villus length compared with that in the SED group and inhibited a high‐intensity running‐induced increase in crypt depth. As shown in Figure 2F–L, the messenger RNA (mRNA) and protein levels of inflammatory cytokines increased in the control group. The TEMPOL‐administered group did not suppress these levels, whereas the RNP^O^ group showed decreased enhancement of inflammatory cytokine mRNA and expressed the protein levels induced by high‐intensity running. To confirm that the suppression of inflammation in the RNP^O^ group was ascribed to intestinal ROS scavenging, the oxidative stress level in the small intestine was evaluated after high‐intensity running with and without antioxidant treatment. Oxidative stress was measured using the protein carbonyl assay and thiobarbituric acid reactive substances (TBARS) assay, which indicate oxidative damage to cellular proteins and membranes, respectively. As shown in Figure 2M, N, oxidative stress of both proteins and lipids increased in the control and TEMPOL groups. In contrast, the high‐intensity running‐induced oxidative stress in proteins and lipids was inhibited in the RNP^O^ group, indicating the remarkable antioxidant effect of RNP^O^ in the GI tract during high‐intensity running. Based on these results, we conclude that intestinal ROS scavenging decreases inflammation, resulting in the suppression of intestinal damage.

### 2.5 RNP^O^ Inhibits the RBC Damage Caused by High‐Intensity Running

**Figure 3:**
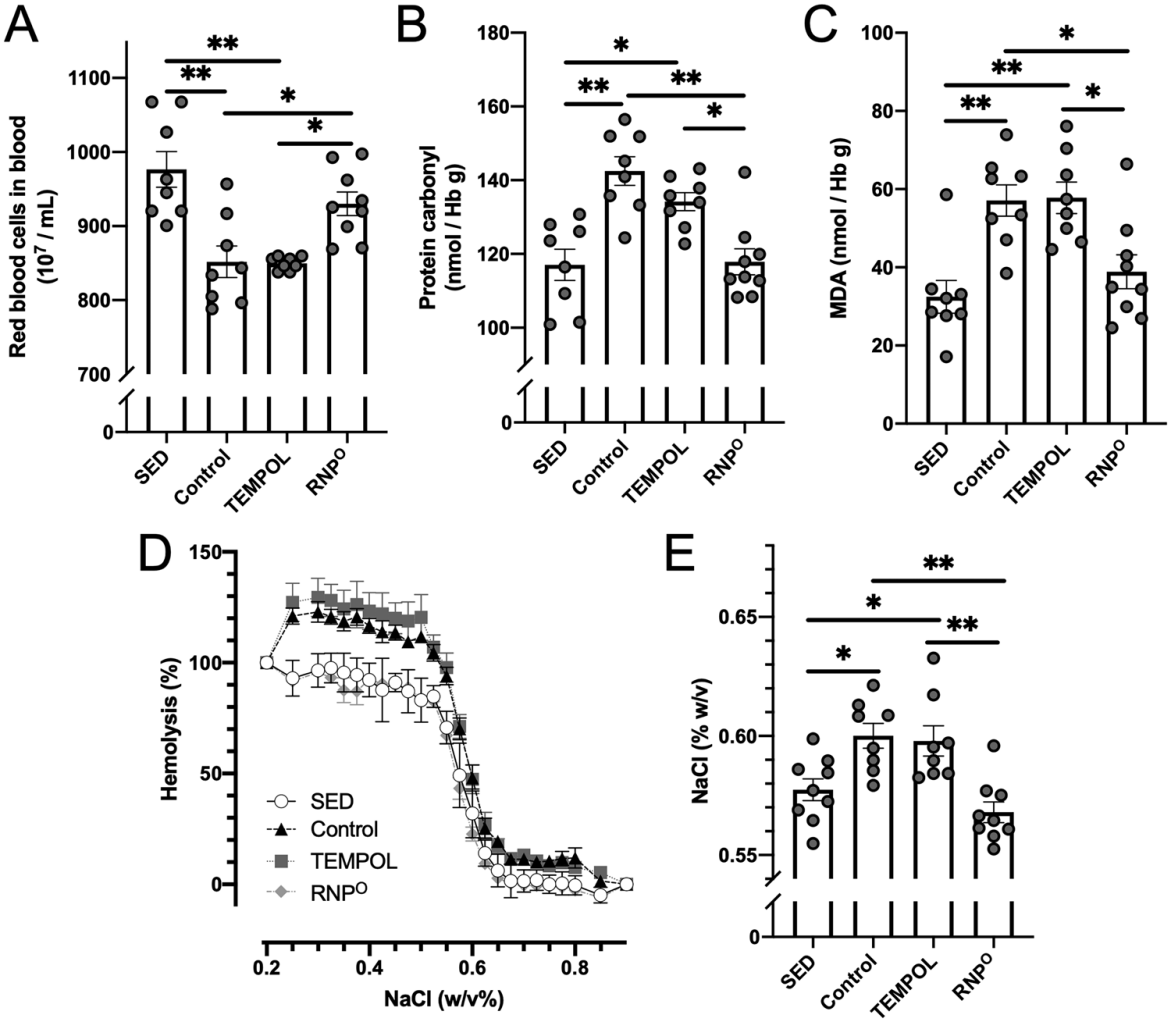
Protection against high‐intensity running‐induced RBC damage by RNP^O^. A) The number of RBCs after rest or high‐intensity running for 80 min (*n* = 8–9). B, C) Oxidative stress levels after rest or high‐intensity running for 80 min, B) Protein carbonyl (*n* = 8–9), C) MDA (*n* = 8–9). D, E) Osmotic fragility of the RBCs collected after rest or high‐intensity running for 80 min, D) Hemolyzed fraction of RBCs in response to NaCl concentration, E) Half‐hemolytic concentrations of NaCl (*n* = 8–9). TEMPOL was administered at 0.69 mmol kg^−1^. RNP^O^ was administered at the same dose as that in the TEMPOL group (0.69 mmol‐TEMPO kg^−1^, 400 mg‐RNP^O^ kg^−1^). Data are expressed as the mean ± SEM (*p < 0.05, **p < 0.01).

As stated above, RNP^O^ inhibits high‐intensity running‐induced oxidative stress, inflammation, and damage to the GI tract. However, further studies are needed to clarify how gastrointestinal dysfunction affects exercise performance. We previously reported that the decrease in RBCs during high‐intensity running is one of the key mechanisms underlying decreased exercise performance.^[^ ^27^ ^]^ That is, the mechanical impact between the soles of the feet and road surface ruptures the RBCs in blood circulation, releasing iron ions, followed by the generation of ROS through a Fenton‐like reaction. Overproduction of ROS further deteriorates circulating RBCs and makes them susceptible to further oxidative stress and mechanical impact. This vicious cycle gradually decreases the number of RBCs in the bloodstream during high‐intensity running. Subcutaneously injected of RNP^N^ is internalized in the bloodstream and circulates for an extended period, followed by protection of RBCs against oxidative damage to inhibit their fragility.^[^ ^27^ ^]^ Orally administered RNP^O^ remains only in the GI tract and eliminates intestinal ROS, as we previously reported.^[^ ^18^, ^21^ ^]^ Therefore, why was exercise performance increased by oral RNP^O^, as shown in Figure 1? To investigate the detailed mechanism by which orally administered RNP^O^ affects exercise performance, we focused on the condition of RBCs after 80 min of running in mice. As shown in **Figure** **3**A, the number of RBCs was significantly decreased in the control‐running and TEMPOL groups than in the SED group, similar to the results of previous reports.^[^ ^27^, ^33^, ^34^ ^]^ Interestingly, the RNP^O^ group did not show a decrease in the number of RBCs in the bloodstream, as in the SED group, even though RNP^O^ was located only in the GI tract. This was further confirmed by the quality of the RBCs after 80 min of running. As shown in Figure 3B,C, the protein carbonyl and MDA levels, which are typical markers of the oxidative stress of intracellular proteins and lipids in the cellular membrane of RBCs, were significantly increased in the control and TEMPOL groups compared to those in the SED group, but not in the RNP^O^ group. The osmotic fragility of RBCs in the RNP^O^ group did not increase compared with that in the control and TEMPOL groups, as shown in Figure 3D,E. For example, the sodium chloride (NaCl) concentration indicating 50% hemolysis of RBC (OF_50_) was significantly increased in the control and TEMPOL groups (control: 0.600, TEMPOL: 0.598), whereas the OF_50_ for the RNP^O^ group was similar to that of the SED group (SED: 0.577, RNP^O^: 0.576). Therefore, these results clearly indicate that intestinal RNP^O^ inhibits oxidative damage and the weakening of RBCs in the bloodstream caused by high‐intensity running.

RNP^O^ was confirmed to protect against damage to RBCs and to affect exercise performance. However, despite its localization in the GI tract, the mechanism by which RNP^O^ exerts a protective effect on RBCs remained unclear.

### 2.6 RNP^O^ Inhibits High‐Intensity Running‐Induced Leaky Gut

**Figure 4:**
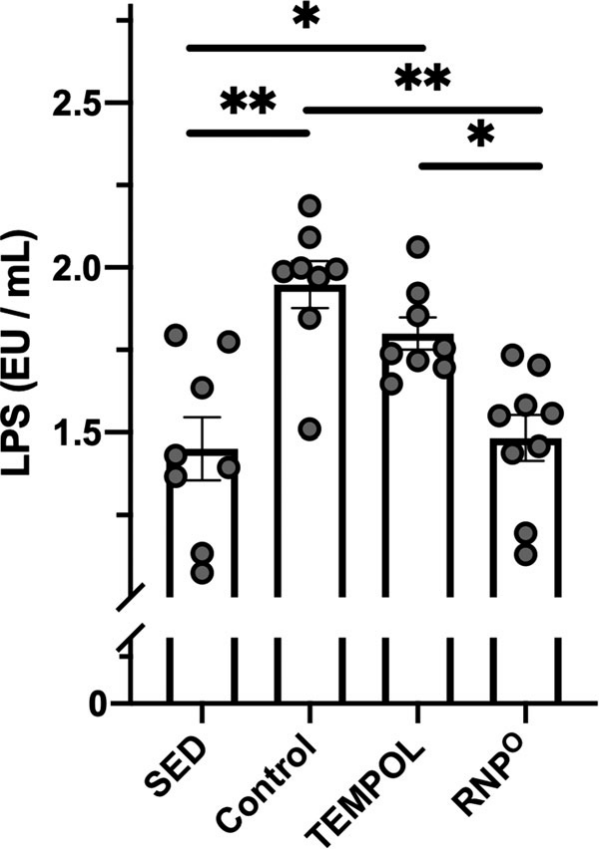
Suppression of high‐intensity running induced‐leaky gut by scavenging ROS in the GI tract. LPS levels in plasma after rest or high‐intensity running for 80 min (*n* = 8–9). TEMPOL was administered at 0.69 mmol kg^−1^. RNP^O^ was administered at the same dose as the TEMPOL group (0.69 mmol‐TEMPO kg^−1^, 400 mg‐RNP^O^ kg^−1^). Data are expressed as the mean ± SEM (*p < 0.05, **p < 0.01).

Elimination of ROS in the GI tract suppresses intestinal inflammation and damage to RBCs, as mentioned above. What is the connecting mechanism between the GI tract and blood? Inflammation in the GI tract, such as ulcerative colitis, is reported to increase the permeability of the intestinal wall, causing substances that remain in the intestine, such as intestinal bacteria, metabolites, allergens, and various toxins, to leak from the intestine into the bloodstream and cause damage throughout the body, known as a leaky gut.^[^ ^35^, ^36^ ^]^ We assumed that the leaky gut might be one of the main factors influencing damage to RBCs from intestinal inflammation. In the context of a leaky gut, various substances such as undigested food particles, toxins and bacterial components, inflammatory mediators, and waste products may potentially leak into the bloodstream and organs that would generally be restricted to the GI tract. Here, we measured the level of lipopolysaccharide (LPS) in plasma as one of the representatives since it is reported at elevated concentrations in the blood by increasing the leakiness of the intestinal wall.^[^ ^37^, ^38^ ^]^ LPS is considered a cause of sepsis and multiple organ failure from the induction of ROS and inflammation. As shown in **Figure** **4**, the plasma LPS level was higher in the control group than in the SED group. The TEMPOL group did not show a decrease in its level. In contrast to these groups, the RNP^O^ group showed clearly suppressed LPS levels, almost similar to those in the SED group, suggesting that suppression of intestinal inflammation by RNP^O^ prevented leakage of the intestinal wall and diffusion of toxic substances into the bloodstream.

### 2.7 RNP^O^ Inhibits the Skeletal Muscle Damage Caused by High‐Intensity Running

**Figure 5:**
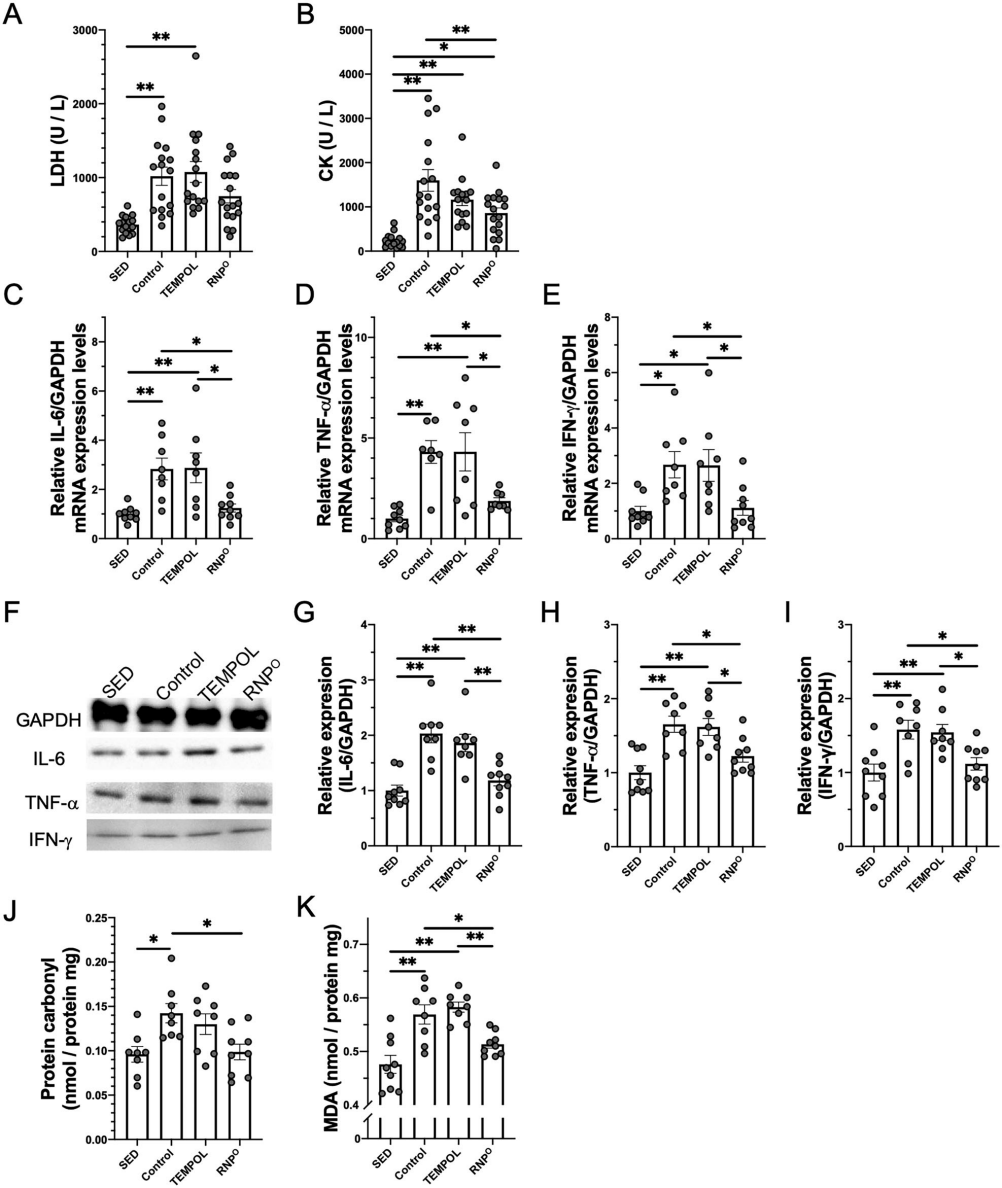
Prevention of high‐intensity running‐induced skeletal muscle damage by suppression of leakiness of gut wall (leaky gut) by oral RNP^O^ administration. A, B) Skeletal muscle damage marker in plasma after rest or high‐intensity running for 80 min, A) LDH (*n* = 16–17), B) CK (*n* = 15–17). C‐E) Gene expression of inflammatory cytokines in the muscle after rest or high‐intensity running for 80 min, C) IL‐6 (*n* = 8–9), D) TNF‐α (*n* = 7–9), E) IFN‐γ (*n* = 8–9). F‐I) Protein expression of inflammatory cytokines in the muscle after rest or high‐intensity running for 80 min, F) Immunoblots of glyceraldehyde‐3‐phosphate dehydrogenase (GAPDH), IL‐6, TNF‐α, and IFN‐γ, G) IL‐6 (*n* = 8–9), H) TNF‐α (*n* = 8–9), I) IFN‐γ (*n* = 8–9). J, K) Oxidative stress levels in the muscle after rest or high‐intensity running for 80 min, J) Protein carbonyl (*n* = 8–9), K) MDA (*n* = 8–9). TEMPOL was administered at a 0.69 mmol kg^−1^. RNP^O^ was administered at the same dose as the TEMPOL group (0.69 mmol‐TEMPO kg^−1^, 400 mg‐RNP^O^ kg^−1^). Data are expressed as mean ± SEM (*p < 0.05, **p < 0.01).

As noted above, the relationship between intestinal ROS elimination and exercise performance may be related to a leaky gut. We have previously shown that high‐intensity exercise also seriously damages peripheral muscles.^[^ ^27^, ^39^ ^]^ Increased oxidative stress due to RBC damage may cause muscle damage. Therefore, we investigated the relationship between intestinal and skeletal muscle damage after high‐intensity running. As we have reported previously, the control and TEMPOL groups showed increased lactate dehydrogenase (LDH) and creatine kinase (CK) levels, which are typical indices of skeletal muscle damage, as shown in **Figure** **5**A,B. In contrast, the RNP^O^ group did not show elevated LDH levels, similar to that in the SED group, and inhibited the high‐intensity running‐induced increase in CK levels. Levels of inflammatory cytokines, such as IL‐6, TNF‐α, and IFN‐γ, in the skeletal muscle were also determined by quantitative polymerase chain reaction (qPCR) and immunoblotting. As shown in Figure 5C–I, the mRNA and protein levels of inflammatory cytokines were increased in the control and TEMPOL groups. However, the RNP^O^ group showed a decrease in the enhanced mRNA and protein levels of inflammatory cytokines induced by high‐intensity running. The protein carbonyl and MDA levels in muscles were then measured to evaluate the oxidative stress caused by running. As shown in Figure 5J, K, protein carbonyl, and MDA levels were increased in the control groups. The TEMPOL group showed no decrease in their levels. However, the RNP^O^ group showed low levels of protein carbonyl and MDA similar to those in the SED group. Therefore, despite its presence in the GI tract, RNP^O^ effectively maintains skeletal muscle function by protecting the GI tract.

### 2.8 RNP^O^ Inhibits the Liver and Kidney Damage Caused by High‐Intensity Running

**Figure 6:**
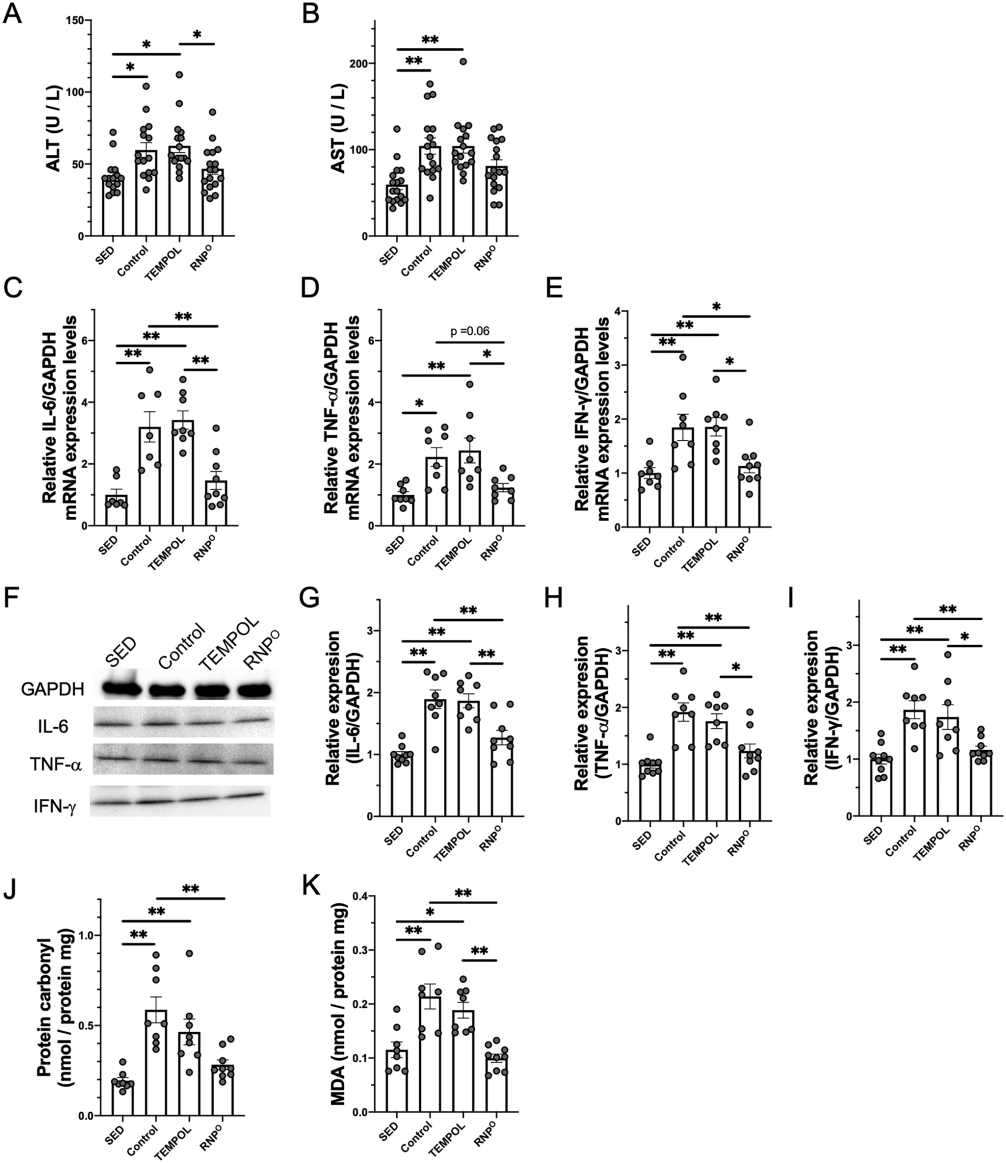
Suppression of high‐intensity running‐induced liver injury caused by EIGS. A, B) Liver damage marker in plasma after rest or high‐intensity running for 80 min, A) ALT (*n* = 15–17), B) AST (*n* = 16–17). C‐E) Gene expression of inflammatory cytokines in the liver after rest or high‐intensity running for 80 min, C) IL‐6 (*n* = 7–9), D) TNF‐α (*n* = 8–9), E) IFN‐γ (*n* = 8–9). F‐I) Protein expression of inflammatory cytokines in the liver after rest or high‐intensity running for 80 min, F) Immunoblots of GAPDH, IL‐6, TNF‐α, and IFN‐γ, G) IL‐6 (*n* = 8–9), H) TNF‐α (*n* = 8–9), I) IFN‐γ (*n* = 8–9). J, K) Oxidative stress levels in the liver after rest or high‐intensity running for 80 min, J) Protein carbonyl (*n* = 8–9), K) MDA (*n* = 8–9). TEMPOL was administered at 0.69 mmol kg^−1^. RNP^O^ was administered at the same dose as the TEMPOL group (0.69 mmol‐TEMPO kg^−1^, 400 mg‐RNP^O^ kg^−1^). Data are expressed as the mean ± SEM (*p < 0.05, **p < 0.01).

Oxidative stress in the GI tract induces intestinal inflammation and a leaky gut. Therefore, toxins are diffused throughout the body, causing damage to normal tissues and peripheral muscles. The liver contributes to exercise performance because of its role in energy metabolism and control of the detoxification system. In fact, Jastrzębski et al. reported liver damage with high‐intensity exercise.^[^ ^40^ ^]^ Therefore, we investigated the effects of RNP^O^ on liver conditions. In our 80 min running experiments, the control group showed elevated levels of alanine transaminase (ALT) and aspartate transaminase (AST), which are known to increase liver damage, as shown in **Figure** **6**A,B. In contrast to the TEMPOL group, which did not show suppression of these levels, the RNP^O^ group did not show increased levels comparable to those in the SED group. High‐intensity running‐related liver damage was also estimated based on inflammatory cytokine levels (IL‐6, TNF‐α, IFN‐γ) by qPCR and immunoblotting. As shown in Figure 6C–I, the mRNA and protein levels of inflammatory cytokines were increased in the control and TEMPOL groups. However, the RNP^O^ group showed a decrease in the enhanced mRNA and protein levels of inflammatory cytokines after high‐intensity running. Lipid oxidation and protein levels were also used to estimate liver damage. As shown in Figure 6J,K, the protein carbonyl and MDA levels were increased in the control and TEMPOL groups, whereas these levels did not increase in the RNP^O^ group, remaining as low as those in the SED group. Blood urea nitrogen (BUN) and creatinine (CRE) levels also increased in the control and TEMPOL groups, as shown in Figure S4, Supporting Information, indicating that high‐intensity running‐induced damage influenced the kidneys, similar to a previous report.^[^ ^41^ ^]^ RNP^O^ treatment clearly decreased these levels.

## 3 Discussion

Although an increasing number of people are exercising for health, exercise may worsen health instead of showing improvements.^[^ ^1^, ^42^ ^]^ High‐intensity or prolonged exercise has been reported to cause gastrointestinal syndrome and anemia.^[^ ^32^, ^33^, ^43^ ^]^ Here, we have applied our nanoparticle‐type antioxidant to an exercise‐induced damage model mouse. To study exercise‐induced damage, several animal models have been reported, such as the forced swimming test and voluntary wheel running.^[^ ^44^, ^45^ ^]^ This study employed high‐intensity running as a model because we observed stable gastrointestinal damage caused by running at speeds above the lactate threshold.^[^ ^4^, ^29^, ^30^ ^]^ The intestinal damage was caused by oxidative stress, resulting in the spread of LPS in blood by increasing the leakiness of the intestinal wall (Figure 2 and Figure 4).^[^ ^46^, ^47^ ^]^ This indicates that versatile intestinal substances, such as toxins, allergens, viruses, and bacteria, simultaneously spread to the whole body.^[^ ^36^ ^]^ In particular, LPS is a known promoter of ROS and inflammation,^[^ ^48^ ^]^ which causes oxidative stress in the bloodstream and damages RBCs (Figure 3B,C). In fact, the number of RBCs decreases significantly after high‐intensity running, possibly because of oxidative stress caused by spreading LPS in the bloodstream (Figure 3A). Destruction of RBCs further increases oxidative stress in the bloodstream via the Fenton‐like reaction of the leaked iron ions, which accelerates further damage to RBCs.^[^ ^27^ ^]^ Therefore, the observed vicious cycle increases oxidative stress in the bloodstream. We interpreted that increased oxidative stress in the intestine is the main cause of EIGS, followed by the leakage of the intestinal wall, which spreads intestinal components throughout the body. This is one of the main factors that increase damage to RBCs, skeletal muscles, liver, and kidneys (Figure 3, Figure 5, Figure 6, and Figure S4, Supporting Information).

As the main player in EIGS is intestinal overproduction of ROS, which may cause exercise‐induced dysfunction of cells, organs, and skeletal muscles, application of antioxidants is the primary candidate for recovery. Many types of antioxidants have been reported to inhibit ROS‐induced dysfunction; however, most antioxidants have shown no effect, though side effects have been suggested.^[^ ^10^, ^49^ ^]^ As shown in Figure 1, the administration of TEMPOL, a representative LMW antioxidant, improved high‐intensity running performance slightly at low doses, but not significantly. However, the all‐out running time tended to decrease with an increasing TEMPOL dose. TEMPOL is rapidly eliminated from the body after oral administration.^[^ ^21^ ^]^ If the problem with LMW antioxidants is only rapid excretion from the body, this tendency cannot be explained. Namely, LMW antioxidants are ineffective in removing overproduced ROS owing to rapid excretion but also induce strong adverse effects on normal cells and organs owing to the dysfunction of cellular redox homeostasis, called as the “overdose syndrome” in the exercise field.^[^ ^13^, ^19^, ^27^ ^]^ Based on these results, we need to focus on the following two points to improve antioxidant treatment on exercise performance: 1) suppression of dysfunction in intracellular redox homeostasis and 2) extension of intestinal antioxidant effects.

RNPs are self‐assembling polymeric micelles composed of amphiphilic block copolymers, possessing antioxidant TEMPO as a side chain of the hydrophobic segment of several tens of nanometers. Owing to their size, cellular uptake was decreased significantly compared to that of LMW TEMPOL, which strongly decreased the dysfunction of intracellular redox homeostasis. This was confirmed in zebrafish and mouse experiments.^[^ ^19^, ^26^ ^]^ There have been many publications on drug encapsulation to control drug distribution. However, drug leakage is observed during circulation in vivo, which decreases encapsulation efficiency.^[^ ^50^ ^]^ The leaked drugs also have adverse effects on normal organs. We covalently conjugated the TEMPO moiety to the backbone of the amphiphilic block copolymer, as another important characteristic to avoid the leakage problem in our RNPs. The cytotoxicity of RNP^O^ in vitro also validates this material design, as we have reported previously.^[^ ^16^ ^]^ Even in the covalent conjugation of TEMPO moiety on the polymer backbone, we have confirmed that its radical scavenging ability is still high enough.^[^ ^25^, ^51^ ^]^ Also, RNPs directly eliminate ROS, such as hydroxyl radical, alkyl peroxyl radical, and superoxide anion radical, in vivo.^[^ ^25^ ^]^ Therefore, we succeeded in widening the “therapeutic window”.^[^ ^19^, ^52^ ^]^

Jiang et al. reported that orally administered nanoparticles tend to accumulate in the intestinal mucosa in a size‐dependent manner.^[^ ^53^, ^54^ ^]^ We also confirmed this tendency using commercial polystyrene nanoparticles. In the case of polymeric micelles formed using amphiphilic block copolymers, such as RNPs, accumulation tendency was much higher than that of commercial polystyrene nanoparticles. RNP^O^ with 40 nm diameter accumulates in the colon mucosa at almost two orders of magnitude higher than that of commercial polystyrene nanoparticles of the same size. Core‐shell structure of RNP^O^ with the PEG surface improves dispersion stability, which might prevent aggregation under harsh intestinal conditions.^[^ ^18^, ^21^ ^]^ The small size, even in the GI tract, might increase accumulation and retention in the intestinal mucosa for over 24 h, compared with that of LMW antioxidants, which are eliminated within minutes.^[^ ^18^, ^21^ ^]^ In addition to these characteristics of RNPs, i.e., the wide therapeutic window (less adverse effects) and long retention in the intestinal mucosa, intestine‐selective localization but no internalization in the bloodstream after oral administration of RNP^O^ is another crucial factor as a body‐benign drug. Based on these characteristics, oral administration of RNP^O^ effectively reduces exercise‐induced EIGS.

**Figure 7:**
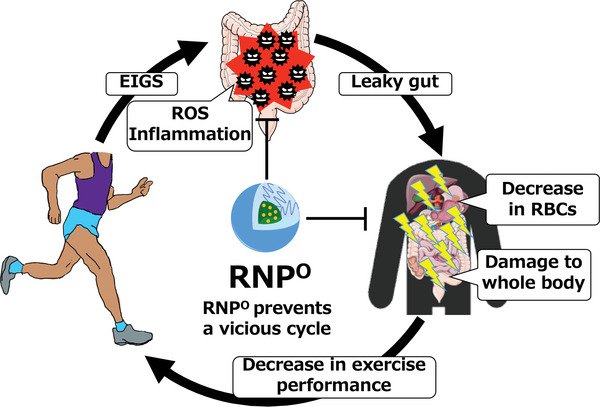
Gastrointestinal ROS impairs systemic function whereas RNP^O^ improves exercise performance.

Our objective was not to elucidate the mechanism of exercise‐induced gastrointestinal inflammation but to determine whether gastrointestinal inflammation is protected by ROS scavenging with our nanoparticle‐type antioxidants, which in turn enhance exercise performance. It is important to note that amelioration of EIGS by effectively eliminating ROS in the GI tract by RNP^O^ is strongly related to exercise performance (Figure 1 and Figure 2). As stated above, leakiness of the intestinal wall (so‐called “leaky gut”) increased owing to inflammation in the GI tract, which was triggered by high‐intensity running (Figure 4). RNP^O^‐induced intestinal ROS scavenging suppressed intestinal inflammation, followed by suppression of leakiness. The level of LPS in the blood was elevated by running, whereas it was decreased by RNP^O^ treatment. It is well known that LPS in blood significantly causes damage to major organs such as liver and kidney.^[^ ^32^, ^55^ ^]^ Here, we confirmed that exercise‐induced damage in inflammation increased damage to the blood, liver, and kidneys (Figure 3, Figure 6, and Figure S4, Supporting Information). The leaky gut also increased damage to the skeletal muscles (Figure 5). Suppression of intestinal inflammation by RNP^O^ treatment inhibited leaky gut, thus preventing damage to the RBCs, skeletal muscles, liver, and kidney. A decrease in RBCs decreases the oxygen supply to peripheral muscles. Based on these results, we concluded that suppression of leaky gut might be one of the most crucial factors for improving exercise performance (**Figure** **7**).

## 4 Conclusion

In conclusion, we have designed a self‐assembling nanoparticle‐type antioxidant, RNP^O^, which selectively localizes in the GI tract after oral administration. Orally administered RNP^O^ remains in the intestinal mucosa for more than one day and continuously eliminates intestinal ROS, which can extend the forced running time by ≈40% on the treadmill (28 m/min). To the best of our knowledge, this is the first demonstration indicating that ROS scavenging in the GI tract improves exercise performance. We have confirmed that high‐intensity running causes intestinal damage (EIGS), followed by leakiness of the intestinal wall (so‐called “leaky gut”), confirmed by the LPS levels in the blood. Therefore, high‐intensity running damages the GI tract as well as the systemic organs, including a decrease in the number of RBCs. Protection of intestinal inflammation by ROS scavenging using RNP° can suppress the leaky gut to prevent systemic organ damage, including RBCs, skeletal muscles, liver, and kidneys, and prolong exercise performance in a dose‐dependent manner. The core‐shell type with several tens of nanometer sizes of RNP^O^ is an essential factor in prolonging retention in the intestinal mucosa and preventing dysfunction of intracellular redox homeostasis, which effectively scavenges ROS from the intestine and enhances exercise performance. Therefore, RNP^O^ is a promising material for enhancing exercise performance and protecting against exercise‐induced organ damage.

## 5 Experimental Section

### Material

PEG monomethyl ether (MeO‐PEG‐OH, number average molecular weight = 5000), lithium bromide (LiBr), ethylenediaminetetraacetic acid (EDTA), and 1, 1, 3, 3‐tetramethoxypropane were purchased from Sigma‐Aldrich, Inc. (USA). Super‐dehydrated DMF, sodium hydride (NaH), and NaCl were purchased from Kanto Chemical Co., Inc. (Japan). The super‐dehydrated solvents were further purified using a solvent purification system (GlassContour, Nikko‐Hansen, Japan). TEMPO, TEMPOL, sodium dodecyl sulfate (SDS), 2,4‐dinitrophenylhydrazone (DNPH), 2‐thiobarbituric acid (TBA), and dibutylhydroxytoluene were purchased from Tokyo Chemical Industry Co., Ltd. (Japan). DMSO, DMF, methanol, 2‐propanol (IPA), deuterated chloroform (CDCl_3_), phenylhydrazine, phosphate‐buffered saline (PBS) powder, 10% formalin neutral buffer solution, tris(hydroxymethyl)aminomethane (Tris), hydrochloric acid (HCl), NP‐40 substitute, sodium deoxycholate, bovine serum albumin (BSA), trichloroacetic acid (TCA), ethanol, ethyl acetate, guanidine hydrochloride, butanol, and pyridine were purchased from FUJIFILM Wako Pure Chemical Industries, Ltd. (Japan). PBS powder was dissolved in dd water and autoclaved for sterilization before use. EDTA disodium salt (EDTA‐2Na) was purchased from Dojindo (Japan). Heparin was purchased from Mochida Pharmaceutical, Inc. (Japan). Saline was purchased from Otsuka Pharmaceutical Co. Ltd. (Japan). Solutions of various NaCl concentrations were prepared using saline and dd water. Skim milk was purchased from Megmilk Snow Brand Co. Ltd. (Japan). Tween‐20 was purchased from Santa Cruz Biotechnology (USA).

### Preparation of RNP^N^

The redox polymer methoxy‐PEG‐*b*‐poly[4‐(2,2,6,6‐tetramethylpiperidine‐1‐oxyl)aminomethylstyrene] (MeO‐PEG‐*b*‐PMNT) was synthesized as described in our previous reports.^[^ ^27^ ^]^ RNP^N^ was prepared via dialysis of MeO‐PEG‐*b*‐PMNT in DMF against dd water.

### Synthesis of MeO‐PEG‐*b*‐PMOT

MeO‐PEG‐*b*‐PMOT was synthesized using the Williamson ether reaction, where the chloromethyl moieties of the side chain in MeO‐PEG‐*b*‐poly(chloromethylstyrene) (MeO‐PEG‐*b*‐PCMS) were reacted with TEMPOL in the presence of NaH in DMF, as previously described (Scheme S1, Supporting Information).^[^ ^23^ ^]^ Briefly, MeO‐PEG‐*b*‐PCMS was synthesized as described in our previous study.^[^ ^27^ ^]^ After MeO‐PEG‐*b*‐PCMS (2.06 g, 0.26 mmol) and TEMPOL (2.51 g, 14.6 mmol) were completely dissolved in super‐dehydrated DMF (10 and 9.2 mL, respectively), they were mixed, followed by adding NaH (0.75 g, 31.3 mmol) and stirring for 24 h at room temperature. After inactivating NaH by adding a small amount of methanol, the reaction mixture was filtered twice to remove precipitates, followed by pouring in cold IPA and centrifuging at −15 °C and 14 660 × g for 10 min. The precipitates were collected, dissolved in warm methanol, and re‐precipitated in cold IPA. This solubilization‐precipitation cycle was repeated five times for purification, followed by overnight drying under reduced pressure to obtain MeO‐PEG‐*b*‐PMOT. The ^1^H NMR spectrum of MeO‐PEG‐*b*‐PMOT in CDCl_3_ was recorded on a JNM‐ECS‐400 spectrometer (JEOL, Tokyo, Japan) at 400 MHz after adding a small amount of phenylhydrazine to reduce the paramagnetic nitroxide radicals. The molecular weight of MeO‐PEG‐*b*‐PMOT was measured by GPC with DMF containing 10 mm LiBr as a carrier at 40 °C (flow rate, 0.6 mL min^−1^) was used with two polystyrene gel columns (TOSHO TSK‐gel α‐M; exclusion limit, 10000,000 Da; particle size,13 µm; 7.8 mm i.d. 30 cm) connected in series with a Jasco PU‐4180 pump, Jasco RI‐2031 refractive index detector, and Jasco UV‐4075 UV detector. The derivatization efficiency of the TEMPO units on the obtained MeO‐PEG‐*b*‐PMOT was analyzed by ESR spectroscopy using a BioSpin X‐band spectrometer (EMXPlus9.5/2.7, Bruker, USA).

### Preparation and Characterization of RNP^O^

RNP^O^ was prepared by dialysis from a DMF (15 mL) solution of MeO‐PEG‐*b*‐PMOT (1.05 g) against dd water (2 L). The MeO‐PEG‐*b*‐PMOT solution was stirred and sonicated for complete dissolution prior to dialysis. A semi‐permeable dialysis membrane with a 3,500 Da molecular weight cutoff (Spectra/Por 3, Repligen, USA) was employed. The dialysate was changed with 2 L of fresh dd water every few hours, and the final dialyzed solution containing RNP^O^ was collected after 48 h. The concentration of nitroxide radicals in the RNP^O^ solution was quantified by ESR spectroscopy using a BioSpin X‐band spectrometer (EMXPlus9.5/2.7, Bruker, USA). The hydrodynamic diameter, zeta potential, polydispersity index, and light scattering intensity of RNP^O^ were characterized by DLS measurements using a Zetasizer Nano ZS (Malvern Instruments, UK).

### Treadmill Running Tests

All animal experiments in this study were conducted based on the animal experiment plan (approval numbers: 19–349, 20–084, and 21–063), inspected, and approved by the Animal Experiment Committee of the University of Tsukuba, Japan.

To investigate the effect of orally administered RNP^N^ on exercise performance, F344 (Fischer 344, ten weeks old, male) rats were prepared and subjected to a high‐intensity running test until they reached their limit and were no longer able to move forward (all‐out test) according to a previous study, except for the administration route.^[^ ^27^ ^]^ TEMPOL or RNP^N^ was administered by free drinking on the day before the all‐out test (≈2.5 mmol‐TEMPO/kg‐weight).

Evaluation of RNP^O^ was performed using nine‐week‐old male mice (C57BL/6J, CLEA Japan, Inc.). The mice were provided with standard chow and water ad libitum and housed under a 10 h/14 h dark‐light cycle with 52.5 ± 12.5% humidity and 23.5 ± 2.5 °C temperature. After one week of acclimatization, all mice were accustomed to forced running on a motorized treadmill (MK‐680, Muromachi Kikai Co., Ltd., Japan) for 20–30 min every day for 5 consecutive days. The mice were then randomly divided into multiple groups and allowed to rest for 24 h. The mice were fasted for 3 h before the running test. Since we have previously confirmed that RNP^O^ retained more than several hours in the intestinal mucosa after oral administration,^[^ ^18^ ^]^ the sample solution (water, TEMPOL, or RNP^O^) was administered orally via sonde 30 min prior to the running test. An all‐out test was performed to assess exercise performance. The all‐out test comprised 5 min of rest on a stationary treadmill, followed by a 5 min warm‐up period during which the speed was gradually increased from 0 m min^−1^ to 28 m min^−1^, the mice were then forced to run at 28 m/min to run to exhaustion. If the mice stopped running and reached the end of the treadmill, they were subjected to mild electrical stimulation to continue running. As the high‐intensity running speed is defined by the lactate threshold (20 m min^−1^ for mice and 28.5 m min^−1^ for rats), we have set above these thresholds (28 and 40 m min^−1^ for mice and rats, respectively) in this study.^[^ ^56^ ^]^ The large range of running times for the all‐out test was inappropriate for biochemical evaluation. Therefore, a different experiment was conducted under the same experimental conditions as the all‐out test, but with a fixed running time of 80 min for biochemical evaluation. Some mice reached their limit earlier than 80 min; therefore, these mice were excluded from biochemical evaluation. Mice in the SED group were treated similarly to those in the 80 min‐running experiments; however, instead of running, they rested on a still treadmill for 80 min after the oral administration of water.

### Blood and Tissue Collection

After the running experiments, the animals were euthanized by whole blood collection under anesthesia by isoflurane inhalation, in which the animals were placed in a sealed container containing isoflurane‐soaked paper. A small amount of EDTA‐2Na (for RBC evaluation) or heparin (for plasma evaluation) was added as an anticoagulant to the blood collected from the inferior vena cava, which was then separated into plasma by centrifugation (10 min, 2000 × g, room temperature). The RBCs obtained as pellets after centrifugation were washed thrice with saline. Some of the washed RBCs were kept on ice immediately after collection, and then stored at 4 °C to measurement of osmotic fragility and for counting the number of RBCs. The remaining washed RBCs were immersed in liquid nitrogen immediately after collection, and then stored at −80 °C for measuring oxidative stress. The duodenum was collected and stored in 10% neutral buffered formalin for histological evaluation. The small intestine, skeletal muscle (gastrocnemius, tibialis anterior, and quadriceps), liver, and kidney were immersed in liquid nitrogen immediately after collection, then stored at −80 °C until biochemical evaluation.

### Histological Evaluation

The duodenum was soaked in 10% phosphate‐buffered formalin, cut into tissue sections (thickness: 5 µm), and stained with H&E. Histological images were captured using a microscope (Keyence BZ‐X710, Keyence, USA). The pictures were randomized by third persons who were blinded to the assignment of the group of pictures. Tissue damage scores were measured and graded according to prior articles as follows^[^ ^57^, ^58^ ^]^: Score 0: normal mucosal villi; Score 1: clear structure, but the visible damage; Score 2: structural elements distinguishable, but divergence of connective tissue identified; Score 3: widespread connective tissue divergence identified; Score 4: damage identified to the extent that structural distinctions are difficult; Score 5: widespread disorganization of villous structure and hemorrhage is evident. To further assess tissue damage, villus length, crypt depth, and the ratios of the villus length and crypt depth were also evaluated.^[^ ^59^ ^]^ Each length was measured using ImageJ software (NIH, USA).

### qPCR Analysis

Total RNA was extracted from the tissues of the small intestine, tibialis anterior muscle, and liver using TRIzol (Thermo Fisher Scientific, USA), followed by measurement of the absorbance at 260 nm using a spectrophotometer (NanoDrop 1000, Thermo Fisher Scientific, USA) to determine the total RNA concentration. The RNA concentration in the samples was adjusted to 40 ng µL^−1^ using Milli‐Q water. Quantitative reverse transcription (RT)‐PCR was performed using the One Step TB Green PrimeScript RT‐PCR Kit II (TaKaRa, Kyoto, Japan) with a Thermal Cycler Dice Real‐Time System (TP‐800; TaKaRa, Kyoto, Japan). The primer sequences used in this study are listed in Table S1, Supporting Information.

### Immunoblot Analysis

Total proteins were extracted from the tissues of the small intestine, liver, and quadriceps muscle samples using radioimmunoprecipitation assay (RIPA) buffer (NP‐40 substitute 1% (v/v), SDS 0.1% (w/v), 1 m Tris‐HCl buffer (pH 8.0) 20 mm, EDTA 5 mm, NaCl 150 mm with phosphatase inhibitor cocktail (Nacalai, Kyoto, Japan), and protease inhibitor cocktail (Nacalai, Kyoto, Japan) in MilliQ), and centrifuged (4 °C, 11000 × g, 10 min) to collect the supernatant. The total protein concentration of each sample was determined using the BCA protein assay kit (Thermo Fisher Scientific, USA). A fixed amount of proteins (small intestine, liver; 20 µg per lane, quadriceps muscle; 7.5 µg per lane) was loaded onto a 12% SDS‐polyacrylamide gel and electrophoresed at 60 V in the stacking gel (≈30 min) and at 120 V in the separating gel (≈90 min); the migrated bands in the gel were then transferred to a poly(vinylidene difluoride) membrane using a Trans‐Blot Turbo Transfer System (BIO‐RAD, Tokyo, Japan) at 25 V for 7 min. Subsequently, the membranes were blocked with 3 or 5% (w/v) skim milk or BSA in PBS with 0.1% Tween 20 (PBS‐T) for 60 min at room temperature. For immunoblotting, the following primary antibodies were used: anti‐β‐actin (ab115777, Abcam, Cambridge, UK, 1/1000, 5% skim milk), anti‐GAPDH (ab181602, Abcam, Cambridge, UK, 1/2000, 5% skim milk), anti‐IL‐6 (ab229381, Abcam, Cambridge, UK, 1/1000, small intestine and liver: 3% skim milk, quadriceps muscle: 3% BSA), anti‐TNF‐α (ab183218, Abcam, Cambridge, UK, 1/1000, small intestine and liver: 3% skim milk, quadriceps muscle: 3% BSA), anti‐IFN‐γ (ab216642, Abcam, Cambridge, UK, 1/1000, small intestine and liver: 5% skim milk, quadriceps muscle: 3% BSA). HRP‐labeled goat anti‐rabbit IgG H&L (HRP) (ab97051, Abcam, Cambridge, UK, 1/10 000) was used as the secondary antibody, and specific proteins were detected by chemiluminescence using a commercial substrate (Clarity Max Western ECL Substrate, BIO‐RAD, Tokyo, Japan). Protein blots were imaged, and band intensities were analyzed using the ChemiDoc XRS+ molecular imager with Image Lab Software (BIO‐RAD, Tokyo, Japan).

### Measurement of Oxidative Stress in Proteins and Lipids

Oxidative damage to several organs and tissues, including the small intestine, RBCs, gastrocnemius muscle, and liver, was analyzed by protein carbonyl and lipid oxidation according to previous reports.^[^ ^27^, ^60^, ^61^ ^]^ The organs were homogenized using TissueLyser LT (QIAGEN, NL) after adding lysis buffer (1m Tris‐HCl buffer (pH:8) 50 mm, NaCl 0.1% (w/v) 150 mm, SDS 0.1% (w/v), NP‐40 substitute 1% (v/v), and sodium deoxycholate 0.5% (v/v) in dd water) adjusted to 100 mg‐tissue mL^−1^ and centrifuged to collect the supernatant (11000 × g, 10 min, 4 °C). The supernatants were used to measure the protein concentration using a Pierce BCA Protein Kit (Thermo Fisher, USA). Protein carbonyl measurements were carried out as follows: 200 µL of homogenates or RBC solution, in which RBCs were washed, followed by 40 times (v/v) dilution with saline, was mixed with 800 µL of 10 mm DNPH in 2.5 m HCl or 800 µL of 2.5 m HCl (for blank). The resulting solution was vortexed and incubated for 1 h on a plate shaker at room temperature in the dark. Subsequently, 250 µL of 50% TCA solution (w/v, dd water) was added to the incubated solution, vortexed, and then centrifuged (20600 × g, 5 min, 4 °C). After discarding the supernatant, the precipitate was washed with 1 mL ethanol and ethyl acetate solution (1:1, v/v) and then centrifuged (20600 × g, 5 min, 4 °C), after which the supernatant was discarded. This washing‐centrifugation cycle was repeated thrice to remove unreacted DNPH. The precipitate was dissolved in 1 mL of a 6 m guanidium chloride solution (in dd water). The solution (200 µL) was then transferred to a 96‐well plate, and absorbance was measured at 370 nm using a spectrophotometer. Protein carbonyls were determined using a molar extinction coefficient of 22 000 m ^−1^ cm^−1^ and the absorbance of DNPH in HCl minus the absorbance of HCl (blank). Protein carbonyl values were expressed as nmol per mg protein determined or nmol per hemoglobin (Hb)‐gram. Hemoglobin levels were measured in the RBC solution as described in our previous article.^[^ ^27^ ^]^

Lipid oxidation was evaluated based on the MDA levels using the TBARS assay. Measurements of the small intestine, gastrocnemius muscle, and liver were performed as follows: 200 µL of the homogenate was mixed with 50 µL of 20% TCA solution (w/w, dd water) and 50 µL of 1% TBA solution (w/w, dd water). The resulting solution was then vortexed and incubated for 1 h at 95°C. After cooling to room temperature, 300 µL of a butanol and pyridine solution (15:1, v/v) was added to the reaction solution. The resulting solution was then vortexed and centrifuged (11 000 × g, 4°C, 10 min). The supernatant (100 µL) was transferred to each well of a 96‐well plate, and the absorbance was measured at 532 nm using a spectrophotometer. As a calibration curve, 1, 1, 3, 3‐tetramethoxypropane, which hydrolyzes to MDA, was used instead of the homogenate. An alternative method was employed for the TBARS assay of RBCs to prevent overlapping hemoglobin absorption at 532 nm.^[^ ^43^ ^]^ Briefly, 40 µL of RBC solution (50 times diluted with PBS, v/v) was mixed with 160 µL of PBS, 5 µL of 40 mm dibutylhydroxytoluene in ethanol, and 100 µL of 30% TCA solution (w/w, dd water). The resulting solution was vortexed and incubated in an ice bath for 2 h. The solution was centrifuged (1000 × g, 4°C, 15 min) and 200 µL of supernatant was separated. The supernatant was then mixed with 15 µL of 0.1 m EDTA (in dd water) and 50 µL of 1% of TBA solution (w/w, dd water). The resulting solution was then incubated for 15 min at 90°C on a plate shaker. The supernatant (100 µL) was then transferred to each of two wells in a 96‐well plate, and the absorbance was measured at 532 nm and 600 nm using a spectrophotometer. The TBARS level was determined using a molar extinction coefficient of 156 000 m ^−1^ cm^−1^ and by subtracting the absorbance at 600 nm from that at 532 nm. In addition, for normalization, hemoglobin concentration was measured according to a previous report.^[^ ^27^ ^]^

### Measurement of Osmotic Fragility

Osmotic fragility tests were performed on the RBC solution according to our previous study to determine the vulnerability of RBCs.^[^ ^26^ ^]^ All measurements were performed on the same day to prevent RBC deterioration.

### Count of RBC

The washed RBCs were diluted 1000 times (v/v) with saline, followed by measurement using a cell counter (Countess II FL Automated Cell Counter, Thermo Fisher Scientific, USA) to determine the number of RBCs. All measurements were performed on the same day to prevent RBC deterioration.

### Determination of LPS

LPS levels in blood were evaluated using the Limulus amebocyte lysate (LAL) assay kit (Pyrochrome with Glucashield Buffer, SEIKAGAKU CORPORATION, Tokyo, Japan). After plasma was collected using an LPS‐free instrument, 50 µL of LAL reaction reagent was added to 50 µL of plasma (10 times diluted by LPS‐free water, v/v), and incubated at 37 °C for 13 min; absorbance at 405 nm was measured. For the calibration curve, a commercial LPS sample was used instead of a plasma sample.

### Biomarker Measurement

The organ function markers such as LDH, CK, ALT, AST, BUN, and CRE, were assessed in 10 µL of plasma samples using an automatic clinical chemical analyzer (FUJI DRI‐CHEM 7000 V).

### Statistical Analyses

All values are expressed as the mean ± SEM. The number of replicates for each experiment and the statistical analysis are indicated in each figure legend. Statistical analysis was performed using GraphPad Prism 8.3.0 software (GraphPad Software Inc., San Diego, CA, USA). Two‐way analysis of variance (ANOVA) with a two‐sided Fisher's least significant difference (LSD) method was used to evaluate the effect of antioxidants (RNP^O^ and TEMPOL) and dose (0.39–0.69 mmol kg^−1^) on the all‐out time. One‐way ANOVA with a two‐sided Tukey's test was used to evaluate the differences between all the compared pairs for the other data. Pearson's correlation coefficient was used to assess the correlation of antioxidants (RNP^O^ and TEMPOL) and their dose (0.39–0.69 mmol kg^−1^) on the all‐out time. The significance level was set at *p* < 0.05 for all tests.

## Conflict of Interest

The University of Tsukuba possesses a patent for this material, which is licensed to CrestecBio, Inc. YN is the advisory and shareholder of CrestecBio, Inc., which holds registered or applying patents on redox nanoparticle. The other authors have no conflict of interest to declare.

## Author Contributions

T.T. designed, performed, and analyzed all the experiments and wrote the manuscript. H.O. and Y.N. contributed to the design and discussion of the experiments and corrected the manuscript.

## Supporting information

Supporting Information

## Data Availability

The data that support the findings of this study are available from the corresponding author upon reasonable request.
