# Supplementary material for: Design of Antioxidant Nanoparticle, which Selectively Locates and Scavenges Reactive Oxygen Species in the Gastrointestinal Tract, Increasing The Running Time of Mice
Source: Adv Sci (Weinh). 2023 Aug 1;10(27):2301159. doi: 10.1002/advs.202301159 (PMC10520625; doi:10.1002/advs.202301159)
Supplement: Supplementary file 1 — Supporting Information [file ADVS-10-2301159-s001.pdf]

ADVANCED SCIENCE
Open Access

Supporting Information

for *Adv. Sci.*, DOI 10.1002/advs.202301159

Design of Antioxidant Nanoparticle, which Selectively Locates and Scavenges Reactive Oxygen Species in the Gastrointestinal Tract, Increasing The Running Time of Mice

*Takuto Toriumi, Hajime Ohmori and Yukio Nagasaki**

Supporting Information

Design of antioxidant nanoparticle, which selectively locates and scavenges reactive oxygen species in the gastrointestinal tract, increasing the running time of mice

*Takuto Toriumi, Hajime Ohmori, Yukio Nagasaki**

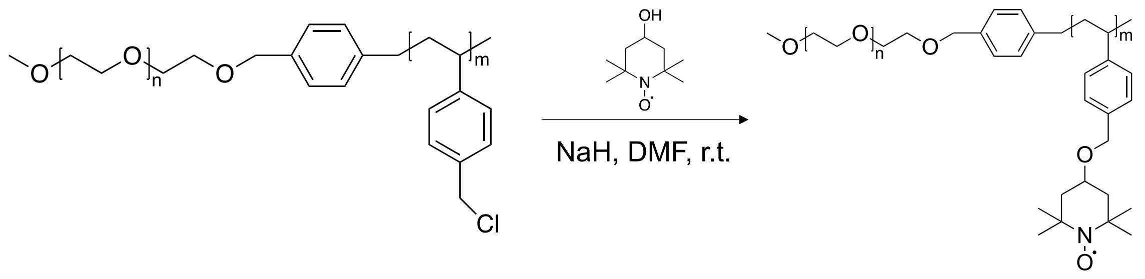

Scheme S1

Synthesis of the methoxy-poly(ethylene glycol)-*b*-poly[4-(2,2,6,6-tetramethylpiperidine-1-oxyl)oxymethylstyrene] (MeO-PEG-*b*-PMOT). (NaH: sodium hydride, DMF: *N,N*-dimethylformamide, r.t.: room temperature)

A

B

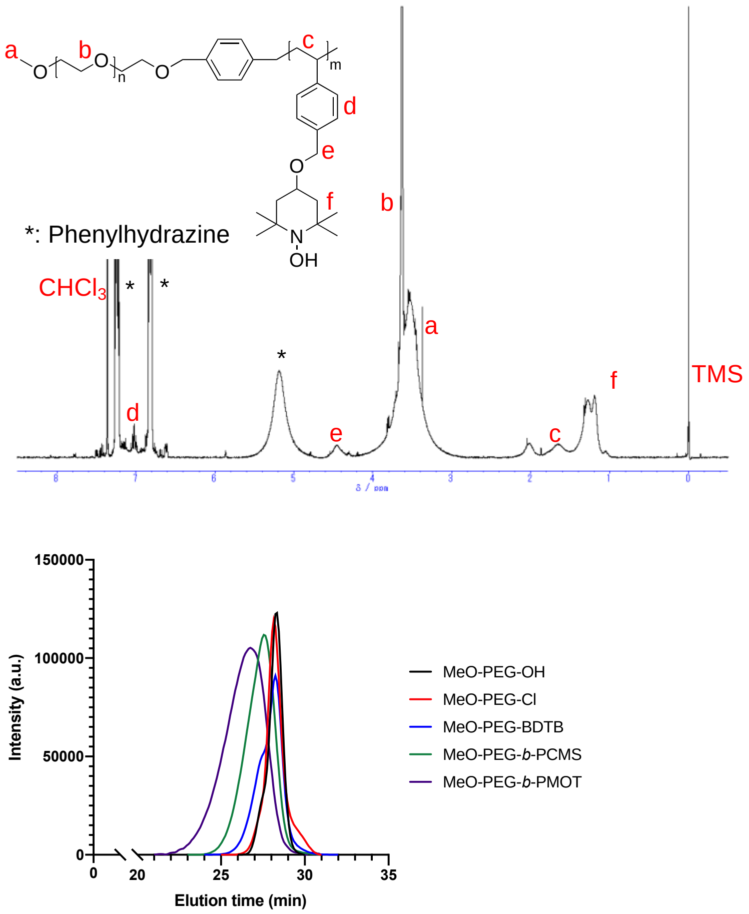

C

	M_n	M_w	M_w/M_n
MeO-PEG-OH	5100	5800	1.14
MeO-PEG-Cl	4400	5800	1.30
MeO-PEG-BDTB	5700	8000	1.41
MeO-PEG-*b*-PCMS	8800	13000	1.47
MeO-PEG-*b*-PMOT	14600	29600	2.03

D

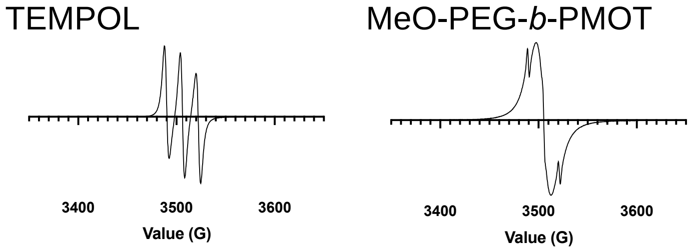

Figure S1

Characterization of MeO-PEG-*b*-PMOT. (A) The ^{1}H-NMR spectrum of MeO-PEG-*b*-PMOT in deuterated chloroform ($CDCl_3$) after reduction with phenylhydrazine, (TMS: tetramethylsilane), (B) Gel permeation chromatograms (GPC) of PEG monomethtoxy ether (MeO-PEG-OH), MeO-PEG-OCH_2PhCH_2Cl (MeO-PEG-Cl), MeO-PEG-$OCH_2PhCH_2SC(=S)Ph$ (MeO-PEG-BDTB), MeO-PEG-*b*-poly(chloromethylstyrene) (MeO-

PEG-*b*-PCMS), and MeO-PEG-*b*-PMOT. (C) List of the number and weight average molecular weights, and the distribution of MeO-PEG-OH, MeO-PEG-Cl, MeO-PEG-BDTB, MeO-PEG-*b*-PCMS and MeO-PEG-*b*-PMOT (M_n means number average molecular weight. M_w means weight average molecular weight.). (D) Electron spin resonance (ESR) spectra of 4-hydroxy-2,2,6,6-tetramethylpiperidine-1-oxyl free radical (TEMPOL) and MeO-PEG-*b*-PMOT in dimethyl sulfoxide (DMSO).

A

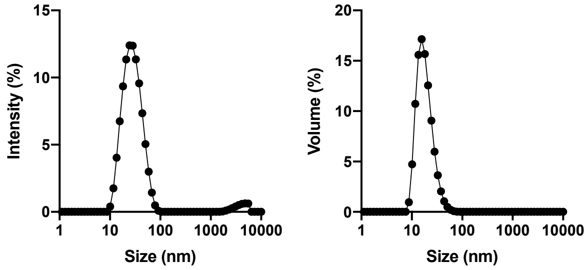

B

	Intensity-Ave. Size (nm)	Volume-Ave. Size (nm)	Zeta potential (mV)	PDI	Scattering int. (kcps)
RNP^O	27±0.6	21±0.6	0.9±0.1	0.22±0.01	32500±240

C

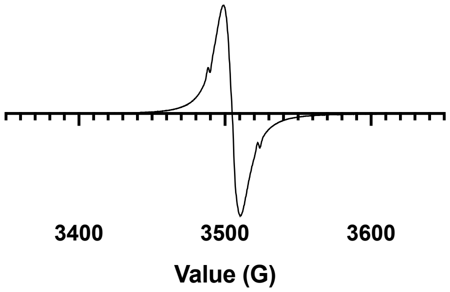

Figure S2

Characterization of the nitroxide radical-containing nanoparticle (RNP^O). (A) The size distribution of the RNP^O analyzed by the dynamic light scattering (DLS) measurement. (B) List of the average size by intensity and volume, zeta potential, polydispersity index (PDI, $\mu\Gamma^{-2}$), and scattering intensity of RNP^O on DLS measurement. (C) ESR spectrum of RNP^O in distilled and deionized (dd) water.

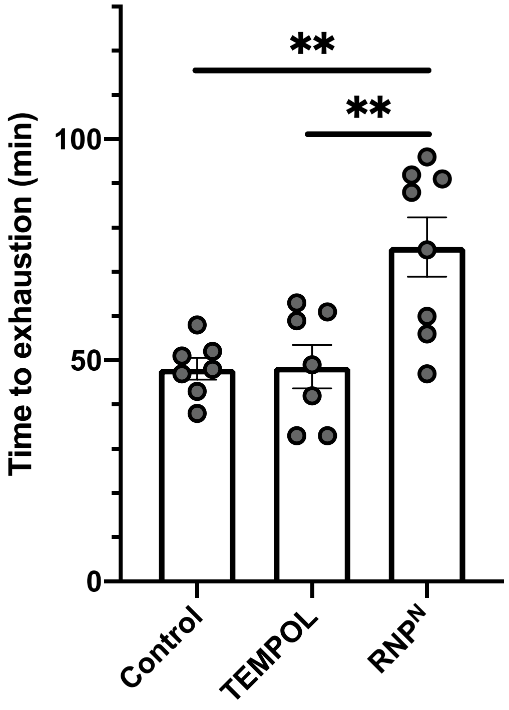

Figure S3

Results of all-out running test in the rat after oral administration of RNP^N. Rats were forced to run until they reached a limit and were no longer able to move forward (all-out test). TEMPOL was administered at approximately 2.5 mmol kg^{-1} body weight. Nanoparticle-type antioxidant (RNP^N, which is pH-sensitive RNP, viz., nanoparticle disintegrates under acidic conditions to expose antioxidant TEMPO outside) was administered at the same dose as the TEMPOL group (approximately 2.5 mmol-TEMPO kg^{-1} body weight, approximately1450 mg-RNP^N kg^{-1} body weight). Data are expressed as the mean ± standard error of the mean (SEM) (n = 7–8) **$p < 0.01$ versus Control (0 mmol nitroxide radicals kg^{-1} body weight) or TEMPOL.

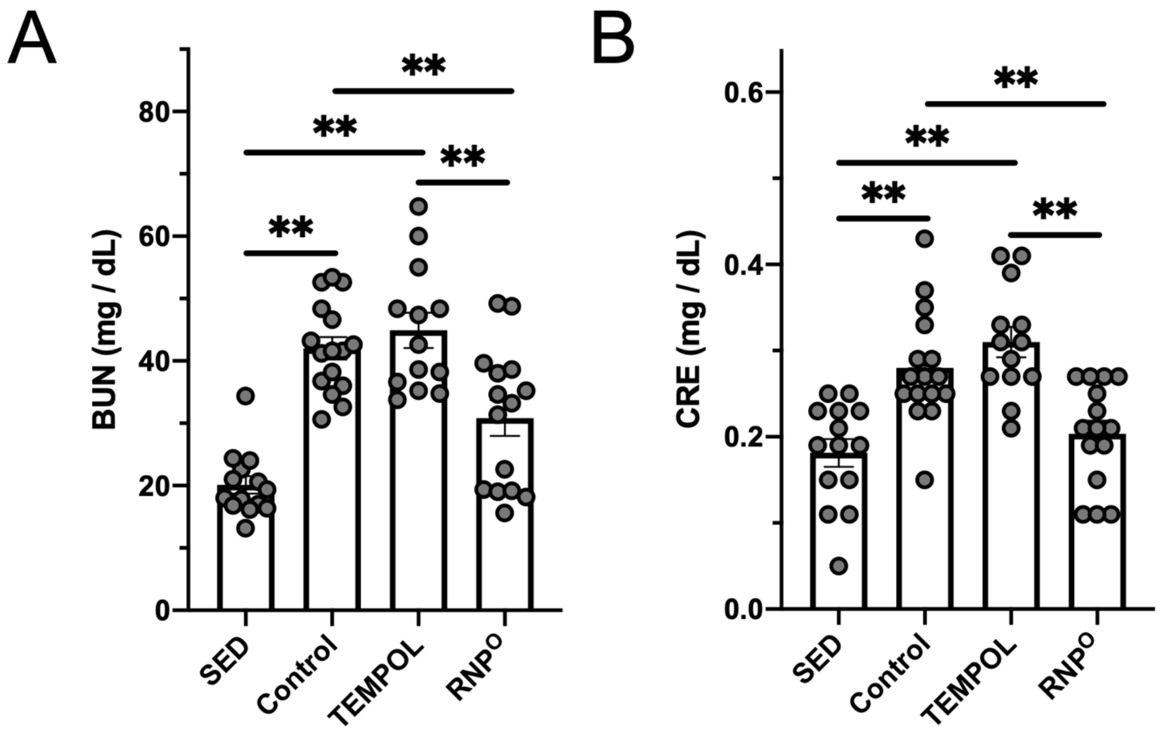

Figure S4

Kidney damage marker in plasma after rest or high-intensity running for 80 min. A) Blood-urea nitrogen (BUN) (n = 13–15), B) Creatinine (CRE) (n = 13–15). TEMPOL was administered at a 0.69 mmol kg^{-1} body weight. RNP^{O} was administered at the same dose as the TEMPOL group (0.69 mmol-TEMPO kg^{-1} body weight, 400 mg-RNP^{O} kg^{-1} body weight). Data are expressed as the mean ± SEM (**$p < 0.01$).

Table S1

List of primers

Primer	Forward (5'-3')	Reverse (5'-3')
b-actin	CTACCTCATGAAGATCCTGACC	CACAGCTTCTCTTTGATGTCAC
GAPDH	GCCATTTGCAGTGGCAAAGTGG	GATGGGCTTCCCGTTGATGACAAGC
IL-6	CTCCCAACAGACCTGTCTATAC	CCATTGCACAACTCTTTTCTCA
TNF-a	ATCCGCGACGTGGAACTG	ACCGCCTGGAGTTCTGGAA
IFN-γ	CAGCAACAGCAAGGCGAAA	CTGGACCTGTGGGTTGTTGAC
